# *Neocosmospora rubicola*, a stem rot disease in potato: Characterization, distribution and management

**DOI:** 10.3389/fmicb.2022.953097

**Published:** 2022-08-11

**Authors:** Muhammad Riaz, Naureen Akhtar, Levini A. Msimbira, Mohammed Antar, Shoaib Ashraf, Salik Nawaz Khan, Donald L. Smith

**Affiliations:** ^1^Department of Plant Pathology, University of the Punjab, Lahore, Pakistan; ^2^Department of Plant Science, McGill University, Montreal, QC, Canada; ^3^Monash Institute of Pharmaceutical Sciences, Monash University, Melbourne, VIC, Australia; ^4^Department of Animal Science, McGill University, Montreal, QC, Canada

**Keywords:** plant growth promoting bacteria (PGPB), fertilizer, potato stem rot, disease control, *Neocosmospora rubicola*

## Abstract

Potato (*Solanum tuberosum* L.) is one of the most important crops in maintaining global food security. Plant stand and yield are affected by production technology, climate, soil type, and biotic factors such as insects and diseases. Numerous fungal diseases including *Neocosmospora rubicola, causing stem rot*, are known to have negative effects on potato growth and yield quality. The pathogen is known to stunt growth and cause leaf yellowing with grayish-black stems. The infectivity of *N. rubicola* across a number of crops indicates the need to search for appropriate management approaches. Synthetic pesticides application is a major method to mitigate almost all potato diseases at this time. However, these pesticides significantly contribute to environmental damage and continuous use leads to pesticide resistance by pathogens. Consumers interest in organic products have influenced agronomists to shift toward the use of biologicals in controlling most pathogens, including *N. rubicola*. This review is an initial effort to carefully examine current and alternative approaches to control *N. rubicola* that are both environmentally safe and ecologically sound. Therefore, this review aims to draw attention to the *N. rubicola* distribution and symptomatology, and sustainable management strategies for potato stem rot disease. Applications of plant growth promoting bacteria (PGPB) as bioformulations with synthetic fertilizers have the potential to increase the tuber yield in both healthy and *N. rubicola* infested soils. Phosphorus and nitrogen applications along with the PGPB can improve plants uptake efficiency and reduce infestation of pathogen leading to increased yield. Therefore, to control *N. rubicola* infestation, with maximum tuber yield benefits, a pre-application of the biofertilizer is shown as a better option, based on the most recent studies. With the current limited information on the disease, precise screening of the available resistant potato cultivars, developing molecular markers for resistance genes against *N. rubicola* will assist to reduce spread and virulence of the pathogen.

## Introduction

Potato (*Solanum tuberosum* L.) ranks as the fourth largest food crop in the world after wheat, rice, and corn (Iqbal et al., 2019; Reyniers et al., 2020). Its world production stands at 376.8 MT per year produced from 19.2 Mha (Tiwari R. K. et al., 2021), with reported pathogens yield losses of up to 17.2% (Savary et al., 2019). If yield losses by pathogens are reduced, potato has the potential to meaningfully assist in achieving the first United Nations Organization’s (Quiroz et al., 2018) development goal of eliminating poverty and malnutrition in the world (Chávez-Dulanto et al., 2021). Recent decades have witnessed a drastic change in the popularity of potatoes in Asian and African countries, which previously depended on other food crops (Tiwari et al., 2021). Therefore, the ever increasing preference of potato consumption necessitates sustainable approaches to ensure the stability of potato production are a must (Kroschel et al., 2020).

Thousands of potato cultivars are available for selection when a farmer decides to engage in production, however, the best selection depends of factors like color, shape, size, texture, cooking quality, starch content and disease resistance (Vilvert et al., 2022). The final decision of cultivar to be produced is further dependent not only on climatic conditions and agronomic practices, but also economic market purpose. Generally, potatoes are prone to a range of diseases that affect the quality and quantity of tuber production starting from the field to the storage facilities. Stem, tuber, and root rots caused by fungi are the most important diseases and cause massive economic losses in potato production (Adolf et al., 2020). *Fusarium* and *Neocosmospora* are among the most important genera of phytopathogens, causing stem rot in the range of crops, potato included (Azil et al., 2021). In genus *Neocosmospora* a pathogen, *N. rubicola* causes stem rot of potato, which is emerging as a serious economically important disease (Riaz et al., 2020). This fungus symptomatically causes stunted growth, yellowing of leaves and typical grayish-black streaking on stems, roots, and tubers. With the spread of pathogen infection, potato plants progressively wilt and then die, causing huge economic losses to the farmers (Riaz et al., 2022).

This review presents various aspects of *N. rubicola* in terms of distribution, symptomatology, and host pathogen interactions, while understanding alterations in type and number of isozymes along with gene expression. On the other hand, integrated disease management (IDM) using potential individual or combined application of plant growth promoting bacteria (PGPB) with commercial fertilizers are discussed in depth. Finally, effects on growth, physiology, and ethylene biosynthesis pathways for potato stem rot disease, which are critical in developing better management approaches, is examined followed by overall conclusions and future perspectives.

## Overview of factors affecting potato production

Several biotic and abiotic stresses, along with relatively limited land allocated for potato cultivation, socioeconomic and management factors play major roles in constraining potato yield.

### Biotic factors

#### Free living pathogenic organisms

Among biotic constraints, insects, viral, bacterial, and fungal pathogens have a drastic impact on the growth and production of potato crops (Demissie, 2019), in particular fungal diseases play a dominant role in affecting potato production (Table 1). However, the survival of fungal pathogens in the absence of potato plants depends upon their ability to deal with unfavorable environments. Most of the fungal pathogens are seed-borne, some of them form resistance structures for their survival, others live as saprophytic organisms on host crop residues or over winter on alternate host plants (Rathore and Shekhawat, 2022). Thus, pathogens acquire ability to directly infect new host species.

**TABLE 1:** Important fungal diseases of potato and their prospective management strategies.

Disease name	Soil/Tuber borne	Causal organism	Management	References
Late blight	Soil and tuber	*Phytophthora infestans*	Certified seed, fungicides, biological and organic amendment	Tiwari I. et al., 2021
Early blight	Soil	*Alternaria solani*	Crop rotation, fungicides, biocontrol agents, proper irrigation, and plant extracts	Yadav et al., 2018
Wart disease	Soil	*Synchytrium endobioticum*	Soil treatment, crop rotation and removal of plant debris	Irshad and Naz, 2013
Stem canker and black scurf	Soil and tuber	*Rhizoctonia solani*	Biocontrol organisms, animal manures, fungicides, and plants extract	Khalil et al., 2019
Powdery scab	Soil and tubers	*Spongospora subterrannea*	Resistance cultivars and cultural practices, antagonistic fungus and bacteria	Sarwar et al., 2018
Pink rot	Soil	*Phytophthora erythroseptica*	Crop rotation, proper drainage, and chemical control	Zhang, 2016
Silver scurf	Soil and tuber	*Helminthosporium solani*	Fungicide seed treatment and proper storage practices	Massana-Codina et al., 2021
Watery wound rot	Soil	*Pythium ultimum or P. debaryamum*	Proper storage and harvesting	Triki et al., 2001
Gangrene	Tuber	*Phoma exigua*	Fungicides treatment, resistant cultivar	Zhao et al., 2021
Dry rot	Soil and tuber	*F. coeruleum, F. eumartii, F. oxysporum and F. sulphureum*	Fungicide and biological control	Jawed et al., 2019
Skin spot	Soil	*Polysecytalum pustulans*	Cultural and fungicide control	Irshad and Naz, 2013
Wilting	Tuber	*Verticillium albo-atrum*	Crop rotation, fungicide control and resistance cultivars	Naraghi et al., 2010
Charcoal rot	Soil	*Macrophomina phaseolina*	Crop rotation, proper nutrition, and drainage	Takooree et al., 2021
Stem rot	Soil and tubers	*Neocosmospora rubicola*	Phytocompounds, PGPB, and nutrition management	Riaz et al., 2020

The most important fungal diseases causing loss of potato production under field conditions and in storage have been the focus of constant effort by Phyto-pathologists attempting to prevent severe yield reductions. Specifically, *N. rubicola* is becoming a pathogen of interest in potatoes as it has been reported to cause losses of up to 20% yield during production (Riaz et al., 2022). Despite current reports on this pathogen its promiscuity among crop species makes it even more alarming. Like most fungal diseases it is favored by environmental conditions and infected seeds.

#### Use of infected and improperly stored seeds

Potato quality is affected by a range of pathogens, especially fungal pathogens, through miscellaneous mechanisms and most importantly through the use of infected seeds. Moreover, post-harvest biodegradation of potato results in losses of about 20–40% worldwide; this is particularly severe in the humid tropical areas which lack suitable harvesting, storage, and processing facilities (Degebasa, 2020). Potato tubers have a delicate outer flesh which facilitates post-harvest entrance of pathogens, particularly when injured. N. rubicola in the presence of high relative high humidity in the field and during the periods of harvesting negatively influences tuber storage (Riaz et al., 2022). Hence, potato tubers stored at high humidity become susceptible to microbial attack that causes high production losses and poor nutritional quality of the potatoes (Wasilewska-Nascimento et al., 2020).

### Abiotic factors

Climate change has negative impact on crop production through the shift of growing seasons which ultimately change life cycles of crops pathogens leading to severe infections (Hunjan and Lore, 2020). The major climate factors which influence plant disease severity and spread include elevated CO_2_, heavy and unseasonal rains, higher humidity, drought, cyclones and hurricanes, and elevated temperature (Mozaffari, 2022). Any shift in climate regimes might change the physiology of pathogen and host plant resistance to infections and the efficacy of plant protection as well as yield ranges. Higher temperatures for instance are located in higher latitudes leading to longer growing seasons and higher yields, but also an increase in pest and pathogen pressure, as a result of more sources of initial inoculum (e.g., infected tubers that survive winter), more vector activity, higher multiplication rates, and more generations per season (Quiroz et al., 2018). Furthermore, temperature affects crop phenology a modification with possible changes in plant-pathogens interactions and new risks of pathogens interferences of both natural and implemented biological control processes (Skendžić et al., 2021). Furthermore, changes in the rate and intensity of extreme climatic events will affect infestations (Naz et al., 2022).

Elevated CO_2_ levels exert affects crop defense systems leading to an increased vulnerability to pathogens (Zhou et al., 2019). The elevated CO_2_ coupled with high temperatures disrupt plant growth and yield (Raza et al., 2019). Increases in the frequency of extreme events (temperature, precipitations, etc.) as well as differences in the sensitivity of higher tropical levels to climatic variability can disrupt the synchrony between the growth, development and reproduction of biological control agents and their hosts (fungal pathogen), leading to perturb ecosystems and increase their vulnerability to invasions through provision of new opportunities for dispersal and growth of pathogens species (Quiroz et al., 2018).

Long-term drought may also lead to reduced crop growth and health thereby increasing their susceptibility to pathogens (Gamalero and Glick, 2022). The synchronization of high humidity and/or high temperature with pathogen-host plant features may enable the presence of other fungus (e.g., early blight caused by *Alternaria solani*, black dot caused by *Colletotrichum coccodes*) and bacterial diseases (Quiroz et al., 2018). In addition to these, some factors indirectly affect potato productivity such as photoperiod, solar radiation, nutrient availability, water use efficiency and natural hazards such as heat waves and night frosts (Nyawade et al., 2019). Soil abiotic constituents such as texture, pH, organic matter content in conjunction with moisture and temperature also greatly affect the behavior of pathogens and determine disease incidence or severity (Fiers et al., 2012).

#### Favorable environmental conditions for potato stem rot

Incidence of stem rot of potato depends upon the virulence of the fungal species involved in the infection, soil, environmental conditions, and potato genotype/cultivar. Potato tubers stored at high humidity and temperature ranging between 15 and 20^°^C develop dry rot infection more rapidly. However, temperature below 5^°^C is an inhibitory factor for fungal growth (Singh et al., 2017). Warmer weather can also diminish resistance mechanisms rendering them inefficient. In tomato, hot weather results in a complete failure of resistance to bacterial wilt and in potato several diseases are activated (Ganiyu et al., 2020). Thus, increase in temperature influences not only the pathogenic microbes and vectors but also affects the resistance mechanisms of the host. It has also been demonstrated, in a range of investigations, that inoculum of *Neocosmospora* spp. survive in soil across a wide range of pH and remain viable in the field for 5–6 years without potato cultivation (Wenham, 2018).

## Potato stem rot by *Neocosmospora rubicola*

Apart from major phytopathogens, numerous fungal, viral, and bacterial diseases are currently gaining importance due to climate change, customer preference and extraordinary demand of spot and rot free potato tubers. Climate modification is a serious threat for humans and primarily affects agricultural ecosystems through increase in atmospheric temperature along with CO_2_ concentration. These changes affect the growth and cultivation of most crops in the world. As a result, some pathogens and diseases become more severe or more prevalent in specific areas of the world, while others decline in importance. Skin spots or blemish diseases affect the quality of tubers and cause withering which becomes important in marketable potatoes. *N. rubicola* is an emerging pathogen causing stem, root and tuber rot diseases in a number of crops, including potato. The disease has gained major attention of the research community worldwide (Kim et al., 2017; Tang et al., 2017; Zheng et al., 2018; Riaz et al., 2020; Arrieta-Guerra et al., 2021). This pathogen is globally distributed and has enormous commercial importance due to its prevalence in the field as well as under storage conditions. It has been reported every year on different crops and is increasing at an alarming rate, and has recently been identified in potatoes (Sepehrnush et al., 2018; Riaz et al., 2022).

### *Neocosmospora rubicola* morphological characteristics

**FIGURE 1:**
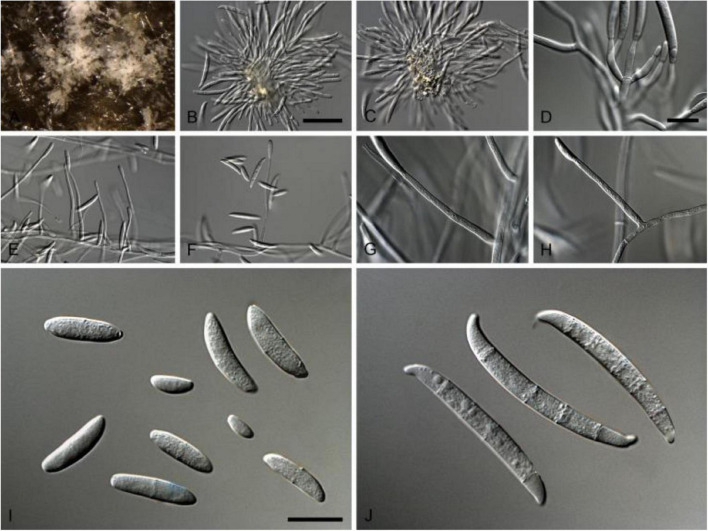
*Neocosmospora rubicola* (ex-type CBS 101018). **(A–C)** Sporodochial conidiophores. **(D)** Conidiogenous apparatus with cylindrical to allantoid phialides. **(E–H)** Simple conidiophores. **(I)** Microconidia. **(J)** Macroconidia. Scale bars: **(B)** 50 μm [apply to **(C,E,F)**]; **(D)** 10 μm [apply to **(G–H)**]; **(I)** 10 μm [apply to **(J)**]. Adapted from Lombard et al. (2015).

This pathogen is a member of fungal family *Nectriaceae* (order *Hypocreales*) of *Phylum Ascomycota*, which includes several serious plant and human pathogens. This family includes around 55 genera that were originally categorized based on their asexual or sexual dimorphs. Members of the family *Nectriaceae* exhibit common phenotypic characters such as uniloculate ascomata that are white, yellow to orange-red or purple in color with phialidic asexual morphs producing amerosporous to phragmosporous conidia (Lombard et al., 2015). Comprehensive morphological characteristics of *N. rubicola* are given in Table 2 and Figure 1.

**TABLE 2:** Morphological characteristics of *N. rubicola*.

Characteristics	*Neocosmospora rubicola*
Colony	Colony on PDA reaching 35–40 mm after 7 days at 24^°^C, forming abundant white to pale luteous aerial mycelium, arranged in concentric rings, richly sporulating on the aerial mycelium; reverse concolorous
*Conidiophores*	*Conidiophores* are mononematous, simple, unbranched, or aggregated into sporodochia.
*Mononematous conidiophores*	*Mononematous conidiophores* 13–129 μm long, 3–7 μm at the base, hyaline, aseptate or septate, terminating in a single phialide or a penicillate or verticillate arrangement of 2–4 phialides; single phialides 17–60 × 3–5 μm, cylindrical, tapering toward the apex, with periclinal thickening and slightly flared collarette; penicillate or verticillate phialides, 13–43 × 3–4 μm, cylindrical to allantoid, tapering toward the apex, with periclinal thickening and slightly flared collarette.
*Sporodochial conidiophores*	*Sporodochial conidiophores* irregularly branched, sometimes slightly stipitate; Sporodochial phialides cylindrical to allantoid, tapering toward the apex, 11–25 × 3–4 μm, with periclinal thickening, with or without slightly flared collarette
*Microconidia*	*Microconidia* mostly produced by mononematous conidiophores, 0–1 (–2)-septate; 0-septate microconidia ellipsoidal to fusiform or obovoid, (8–)9–13 (–19) × (2–)3–4 (–5) μm (av. 11 × 4 μm); 1-septate microconidia, ellipsoidal to fusiform, straight to slightly curved, apex acutely rounded, base sometime flattened (13–)15–20 (–22) × (3–)4–6 μm (av. 18 × 5 μm); 2-septate microconidia rarely formed, ellipsoidal to fusiform, straight to slightly curved, 20–22 (–24) × 4–6 μm (av. 22 × 5 μm).
*Macroconidia*	*Macroconidia* 3–5-septate, cylindrical, straight, or curving at both ends, beaked at both ends: 3-septate macroconidia (27–)32–44 (–47) × 4–6 μm (av. 38 × 5 μm); 4-septate macroconidia (35–)38–48 (–53) × 4–6 μm (av. 43 × 5 μm); 5-septate macroconidia (44–)45–49 (–51) × 5–6 μm (av. 47 × 5 μm).
*Chlamydospores*	*Chlamydospores* not observed

Originally described by Lombard et al. (2015).

### Genetic variability in the genus *Neocosmospora*

The genus *Neocosmospora* comprises of approximately 900 species that are reported to infect nearly 100 plant families. Species of this genus is ubiquitous and survive mostly on living as well as dead plants, soil, air, and water. Earlier, the species of *Neocosmospora* were included in the genus *Fusarium.* However, Nalim et al. (2011) reclassified them from the *Fusarium solani* group as members of *Neocosmospora*. Later, based on DNA sequence data from several loci [Actin (Act), Internal transcribed spacers (ITS), Large subunit ribosomal ribonucleic acid (LSU rRNA), RNA polymerase subunit I (rpb1), Translation elongation factor 1-alpha (tef1), and beta-tubulin (β-tubulin)], with integrated morphological characterizations, *Neocosmospora* was moved as a separate genus in the family *Nectriaceae* (Hirooka et al., 2012; Sandoval-Denis et al., 2019). Hsieh et al. (2020) also included *N. rubicola* in their paper entitled “First Inventory of fungi in symptomless and symptomatic Chinese Mesona indicates Phyto pathological threat” as an emerging pathological threat to the agriculture sector.

### Plants affected by *Neocosmospora rubicola* and their symptoms

**FIGURE 2:**
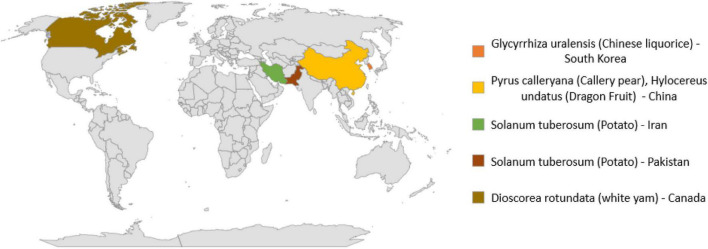
Global distribution of *Neocosmospora rubicola* and host plants.

In addition to human and animal pathogens, *N. rubicola* is causative agent of many crop and ornamental plants diseases as given in Table 3 and Figure 2. This fungus causes stunted growth, yellowing of leaves and typical grayish-black streaking or spots on stems, roots, and tubers. Symptomatic infected potato plants initially showed water-soaked brown to black lesions on the lower stems, near the collar. With the spread of infection, potato plants gradually wilted and then died (Riaz et al., 2020).

**TABLE 3:** Global distribution of *Neocosmospora rubicola* and their host plants.

Pathogen	Crops	Disease	Country	References
*N. rubicola*	*Glycyrrhiza uralensis*	Root rot	Korea	Kim et al., 2017
*N. rubicola*	*Pyrus calleryana*	Root rot	China	Tang et al., 2017
*N. rubicola*	*Hylocereus undatus*	Stem rot	China	Zheng et al., 2018
*N. rubicola*	*Solanum tuberosum*	Associated with infected tubers, crown, and roots	Iran	Sepehrnush et al., 2018
*N. rubicola*	*Dioscorea rotundata*	Dry rot	Canada	Arrieta-Guerra et al., 2021
*N. rubicola*	*Solanum tuberosum*	Stem rot	Pakistan	Riaz et al., 2020; Riaz et al., 2022

### Different mycotoxins produced by *Neocosmospora rubicola*

*Neocosmospora rubicola* is a relatively new threat and to date very little data is available on the mycotoxins the pathogen produces. Various species of *Neocosmospora* group are sporadically associated with mycotoxicosis (Torres and Kontoyiannis, 2011; Klomchit et al., 2021). Being toxin producers, they have shown a wide range of toxic activities against plants. The list of known toxic metabolites produced by *Neocosmospora* include furanoterpenoids (promotes the necrotic activity), ipomeanols (interfere with normal cell growth), ipomeanine (damaged sweet potato cells), and naphthazarins (effects on the cytology of leaves) (Sandoval-Denis et al., 2019; Klomchit et al., 2021). Matkawala et al. (2019) reported that some species of *Neocosmospora* produce trypsin-like serine proteases that enhance its pathogenicity. Hydrolases and proteases are required for the growth and survival of pathogens and also elicit host defense responses in plants (Lu et al., 2020). Based on the limited information regarding *N. rubicola* related to potato it is reasonable to think they produce mycotoxins that aid in infecting their host plants.

## Transmissibility, diagnostics, and management options

### Mechanisms by with *Neocosmospora rubicola* infect host plants

Currently, there is limited information available on the mechanism of action of this pathogen. However, an *in vitro* study by Riaz et al. (2020); demonstrated that *N. rubicola* is a serious threat to potato cultivation, causing stem rot through injuries or lenticels in the absence of wounds. The hyphae of *N. rubicola* grow intracellularly at the initial stage of infection and become intercellular in dead cells. Potato tubers during stem rot infection, undergo numerous biochemical changes that induce biosynthesis of phenolic acids which then converts into lignin or cross-linked cell walls (Chourasia et al., 2021). Association and aggressiveness of *N. rubicola* rot to plants differs between geographic regions including climatic conditions and location of a field (Riaz et al., 2022). *Neocosmospora rubicola* spores remain viable in the soil and on infected potato debris for a long period in the absence of a host plant. The survived spores in the soil cause rotting in progeny tubers and stems after wounds developed due to intercultural operations (Riaz et al., 2020). This results in the yield loss and the build-up of infectious inoculum spores implying more disease pressure for the following seasons.

### Primary diagnostic tools used for identification of *Neocosmospora rubicola* in potato

The primary step considered essential to establish any management strategy against stem rot pathogens is the use of diagnostic methods that provide correct identification and quantification of these pathogens. Phenotypic methods for identifying pathogens rely on relatively few morphological and cultural characters. Therefore, current phylogenetic studies play an important role in the identification and differentiation of closely related strains of pathogens (Guarnaccia et al., 2019). In recent years, several diagnostic techniques, such as DNA sequencing, have been employed for the detection of pathogens associated with potato diseases. The intervening of internal transcribed spacers (ITS) in regions of rDNA have become the most popular marker used to classify fungi into taxonomic groups (Schoch et al., 2012; Yang et al., 2018; Li et al., 2020). Zheng et al. (2018) also did this based on morphological characteristics and amplification of these gene regions in fungal isolates identified as *N. rubicola.*

## Management approaches for potato stem rot caused by *Neocosmospora rubicola*

### Potato varieties

In most countries the use of genetically modified organisms (GMO) through transgenesis are restricted by law which limits the use of resistant varieties that would otherwise be alternative options to suppress pathogen spread and disease severity. Plant resistance is either systemically acquired or induced where both stimulate plant immune response to pathogens equally. It involves activation of the plant immune system through elicitors or natural molecules that suppress the pathogens attack *via* microbes (Yu et al., 2019). Hence, key factors of host resistance require the plant immune system to respond, which involves either recognition of pathogen-associated molecular pattern (PAMP) or by plant pattern recognition receptors (PRR), referred to as pattern triggered immunity (PTI) (Noman et al., 2019). PAMPS are highly conserved molecular components which are present in the pathogen as multiple bacterial cell-surface compounds (flagelin, peptido glycan, and lipopolysaccharides), and major cell wall constituents of higher fungi (Ranf, 2017). *N. rubicola* produces anti-pathogen compounds currently known to include; Aromadendrene, Penicidin, Ethyl p-methoxycinnamate, 2-Coumaranone, and 2-Methyl resorcinol, any or all of which may play a role in pathogenicity by altering the host plant resistance (Wagh et al., 2022).

### Rhizosphere management

In relation to plant essential nutrients, two approaches can contribute to achievement of this goal; (i) the development of new potato varieties for more effective nutrient uptake, (ii) effective management of the rhizosphere (Romera et al., 2019). The rhizosphere and associated soil volume are determined by root systems of plants which act as key energy source permitting development of populations of numerous specific microbes (Pii et al., 2015). Some of these microbes are phytopathogens that cause diseases and, as such, negatively influence plant development. However, most of the rhizobacteria and fungi are advantageous for plants as they promote plant growth and also help the plant (or the total holobiont) adapt to stressful conditions (Kundan et al., 2015). These rhizosphere microbes establish mutualistic associations with plant roots that help in provision of efficient phosphorous and nitrogen nutrition (Wipf et al., 2019). The microbes in association with plant roots produce nutrient solubilizing compounds that liberate bound nutrients or sometimes alter root physiology of plants (Pathak et al., 2019). Some rhizosphere microorganisms inhibit rhizosphere processes and negatively affect plant growth.

## Plant based management approaches to control potato stem rot caused by *Neocosmospora rubicola*

### Host resistance and pathogens recognition after the invasion

Once a pathogen invades the host and successfully suppress PTI, the plants rely on a second layer of more specific recognition of microbes which is encoded by another resistance protein (effector-triggered immunity- ETI). It is based on intracellular immune receptors that are mostly nucleotide-binding site, leucinerich repeat (NBS-LRR) proteins. These proteins can confer more durable and robust resistance (Peng et al., 2018; Yoo et al., 2020; Yuan et al., 2021). Pathogen recognition by ETI leads to hypersensitive responses upon pathogen attack related to the site and results in programmed cell death by rapid ethylene (ET) production. Ethylene induction differs in secretion and activation of defense related phytohormones from Jasmonic acid (JA) and Salicylic acid (SA). This activation of ET plays an important role in the defense signaling network for regulating effective broad-spectrum resistance in host plant (Bian et al., 2020). To date there is limited information on the mechanisms of action of *N. rubicola* and this is not the focus of this review.

### Host-microbiome interactions

A plant, with its associated microbes, forms a holobiont. The plant role in the holobiont is to act as host, and provide niche for growth and proliferation of the diverse associated plant microbiota/phytomicrobiome (Cregger et al., 2018; Ma et al., 2021). The interaction between members of the holobiont enhance plant fitness through improvement of availability of mineral nutrients and modulation of phytohormones level which increase plant productivity (Berlanga-Clavero et al., 2020). The plant holobiont recruits microbial taxa with biocontrol properties to suppress phytopathogens (Backer et al., 2018; John et al., 2020; Lu et al., 2021; Lyu et al., 2021). Plants employ complex mechanisms to suppress phytopathogens which include structural modifications, exudation of specific metabolites and coordination of action in various defense responses (Olanrewaju et al., 2019; Pascale et al., 2020; Wang et al., 2021). Taken together the mechanisms of plant-microbe interaction result into induced systematic resistance to pathogens.

### The plant broad-spectrum resistance

Jasmonic acid coordinates defense responses against necrotrophic pathogens and chewing insects whereas, salicylic acid defense is mainly linked against biotrophic pathogens. The recognition of pathogen elicitors also involves in the synthesis and transport of various lytic enzymes and toxic substances to suppress the spread of pathogens. Therefore, these defense responses in plants exhibit their resistance based on the nature of stimuli and plant regulatory pathways. The route of mechanism for this defense system can be differentiated by systemic acquired resistance (SAR) and induced systemic resistance (ISR) (Langridge, 2017; Wani et al., 2018; Romera et al., 2019; Kamle et al., 2020; Maithani et al., 2021). The appropriate stimulation of SAR and ISR enhance defensive capacity of the host plant against various pathogens. The colonization of growth promoting rhizobacteria and mycorrhizal fungi regulate the stimulation of ISR creating resistance in plants against the attack of phytopathogens (Mashabela et al., 2022), resistance phenomenon referred to as priming. Whereas SAR resistance is induced throughout the plant after exposure to virulent or avirulent pathogenic microbes (Sharma et al., 2019; El-Maraghy et al., 2020; dos Santos, 2021).

Studies on several plant models revealed the resistance system in plants which is generally explained by the production of pathogen-related proteins during regulatory signaling pathways (Rashid and Chung, 2017). Once an outbreak occurs, the magnitude of the plant response is enhanced and disease is suppressed (Xin et al., 2018). Due to broad-spectrum of effectiveness of PR proteins, rhizobacteria-mediated ISR resistance is considered mechanically similar to pathogen-induced SAR. Unlike SAR, R-ISR does not necessarily involve plant defense responses, induced by salicylic acid (Lösera et al., 2021). It has been verified that SA is a key signaling molecule in both locally and systemically induced resistance responses (Antar et al., 2021). However, ethylene plays an important role in rhizobacteria mediated ISR (Singh et al., 2022). A wide range of microbes use the strategy of biocontrol agents that trigger ISR in plants, confirmed their ability to initiate or enhance in the production of plant defense enzymes such as; polyphenol oxidase (PPO), peroxidase and phenylalanine ammonia lyase (Kannojia et al., 2019; Antar et al., 2021; Santoyo et al., 2021). Several studies on molecular mechanisms revealed that PGPR can be used against several pathogens to induce priming of volatile compound without using Npr1 (SA insensitive or non-expresser of PR) gene but the expression of ET signaling is required (Tyagi et al., 2018; Panpatte et al., 2020; Abbasi et al., 2021).

Ethylene is the simplest unsaturated hydrocarbon gas molecule and is an important regulator of PTI against biotrophic pathogens. The ethylene pathway is mainly triggered in defense priming with PGPR and acts as primary recognition mechanism between beneficial bacteria and the host plants (Bukhat et al., 2020). Polko and Kieber (2019) determined that the biosynthesis of ET begins with the conversion of methionine to Sadenosyl methionine, which is ultimately modified as 1-aminocyclopropane-1-carboxylic acid (ACC), catalyzed by ACC synthase (ACS) (Jha et al., 2021). After that 5-methylthioadenosine (MTA) is produced by the action of ACS, which is further converted into methionine through a modified methionine cycle (Pattyn et al., 2021). This salvage pathway preserves the methyl group for another round of ethylene production which is synthesized continuously without an increase in the methionine pool, once started (Tiwari et al., 2022). In the final step, ACC is oxidized into ethylene and hydrogen cyanide by the activity of ACC oxidase which is further detoxified to cyanoalanine-by-cyanoalanine synthase (Kubo, 2015). Cyanoalanine synthase prevents the toxicity of accumulated cyanide during high rates of ethylene synthesis (Zia Ul Haq et al., 2020).

Recent studies on sequence analysis revealed that ethylene receptors are divided into two key subfamilies. Arabidopsis plant, ETR1 and ERS1 receptor isoforms are grouped into subfamily-I whereas, ETR2, ERS2, and EIN4 receptor isoforms are placed in subfamily-II (Berleth et al., 2019). This entire group of receptor isoforms can be identified by modular structure of bacterial sensor histidine kinases. With generally similar overall structures individual isoforms contain major differences in these modules. For instance, type-II receptors have an additional fourth transmembrane helix which is thought to function as a targeting signal (Bi and Zhou, 2021).

## Biological control

The massive use of fungicides, now being reconsidered because of environmental concerns and the emergence of resistance against fungicides, has forced the plant scientific community to search for alternatives of fungicides. One such alternative is antagonistic microorganisms which can restrain stem rot causing pathogens including *N. rubicola* (Bojanowski et al., 2013), revealed that the antagonistic microbes including *Pseudomonas*, *Pantoea*, *Enterobacter*, *Bacillus cereus*, *B. cepacia*, *P. fluorescens, Trichoderma harzianum*, and an *arbuscular mycorrhizal* fungi play a major role in the mitigation of potato rot diseases. Some biopesticides, such as Biosave 10LP and 11LP (*Pseudomonas syringae*) are also been registered in the USA to control dry rots and silver scurf in potato (Al-Mughrabi et al., 2016). Recent data reveal that seed priming with *Bacillus subtilis* formulation resulted in an approximately 56% reduction of potato common scab (Al-Mughrabi et al., 2016). Some potato cultivation regions have adopted pentachloronitrobenzene (PCNB) (common name Quintozene, trade name Blocker, AMVAC) soil treatment (in-furrow) to suppress common scab (Braun et al., 2017). In USA chloropicrin is used as a broad-spectrum biocidal fumigant in the soil, to improve the control of soil-borne potato diseases (Webster et al., 2010).

Currently, a combination of three bacterial inoculants (*Azospirillum lipoform*, *Pseudomonas putida*, *Aztobacter crococum*), and chemical fertilizer nutrition in an aeroponic culture system is the most important factor for the quantitative and qualitative production of healthy potato seeds (Nasiri et al., 2022). The combination bacterial formulation (BF) containing strains of *A. chroococcum, A. lipoferum* and *P. putida* has the potential to increase the tuber yield of potato in healthy soil as well as in the soil infested with *N. rubicola* (Riaz et al., 2022).

*Azotobacter chroococcum* is able to solubilize phosphorous and other compounds such as siderophores, indole acetic acid, and hydrocyanic acid (HCN) during stress (Gopalakrishnan et al., 2011). It also promotes generation of phytohormones such as; gibberellins, cytokinin’s, auxins, vitamins, antibacterial, and antifungal compounds (Babalola, 2010; Wani et al., 2016). *Azotobacter chroococcum* inhibited the *Macrophomina phaseolina*, which is the cause charcoal rot of sorghum (Gopalakrishnan et al., 2011), reduced the infectivity of *Fusarium solani* on tomato plants (Muslim et al., 2021), and have the potential to control *Fusarium* dry rot of potatoes (Janisiewicz and Korsten, 2002). This bacterial strain also has high antagonistic capacity against pathogenic fungi like, *F. solani* and *M. phaseolina* causing okra root rot disease (Al-Taie and Abdul–Hdi, 2021). Formulations constituting mixtures of *Azotobacter* and *Azospirillum* strains have the potential to control root rot disease complexes of *M. phaseolina*, *R. solani*, and *F. solani* (El“_Komy et al., 2020).

*Azospirillum lipoferum* can enhance phosphate solubilization, produce plant-growth promoting hormones such as auxins, cytokinin, and gibberellins and siderophores (Seddigui Kiasari et al., 2018). This bacterial strain also produces catechol-type of siderophores during iron-starved situations like; 2,3-dihydroxybenzoic acid (DHBA), 3,5-DHBA and salicylic acid coupled with lysine and threonine. In addition to their role in iron transport, these siderophores displayed antimicrobial effectivity against various plant fungal and bacterial pathogens (Shah et al., 1992). *Azospirillum lipoferum* has the capacity to alleviate inhibition of chickpea growth under the salinity stress (El-Esawi et al., 2019) and improve maize seed germination (Rozier et al., 2019). *Azospirillum lipoferum* suppressed early blight of potato by triggering ISR (Mekonnen and Kibret, 2021). The activities of defense related enzymes (phenylalanine ammonia lyase, peroxidase, and PPO), quantity of total phenolics, and level of transcriptomic PR-gene was significantly improved by *Azospirillum lipoferum*. Furthermore, it improved H_2_O_2_ and salicylic acid levels up to 3.1- and 1.9-fold, respectively, and decreased cell death up to 1.73-fold in potato plants, through antagonism toward the early blight pathogen (Mehmood et al., 2021).

*Pseudomonas putida*, a gram-negative bacterium, provides benefits to the crop plants by acting as a phosphate solubilizer and biocontrol agent (Sun et al., 2017; Mohamed and Farag, 2020). Previous, study revealed that this strain is able to help manage several plant diseases by producing antibiotics, competition with other pathogens for space and nutrients, and stimulation of host systemic resistance (Riaz et al., 2022). This bacterial strain has a Type-VI Secretion System (T6SS) used to kill and eradicate plant pathogens and is vital in its bio-control portfolio (Borrero de Acuña and Bernal, 2021). It was found promising against several destructive plant pathogens such as *Fusarium oxysporum* in tomato, *Rhizoctonia solani* in potato and *Pectobacterium atrosepticum* in potato, *Sclerotinia sclerotiorum* in lettuce and *Ralstonia solanacearum* in tomato (Bernal Guzmán et al., 2017; Durán et al., 2021). Siderophores produced by *P. putida* also improve iron nutrition of crop plants (Meliani et al., 2017). *Pseudomonas putida* BP25 produced 2-ethyl-3,6-dimethylpyrazine and 2-methylpyrazine; that constrained the entire suite of developmental stages of *M. oryzae* such as conidial germination, mycelial growth, and sporulation. It suppressed several pathogens by producing volatile organic compounds related to pyrazine such as 2- ethyl-3,6-dimethylpyrazine and 2-methylpyrazine (Patel et al., 2021).

Consequently, PGPR could be important in potato production, as rhizobacteria lessen the need for application of fertilizer (Aloo et al., 2021). Rhizobacteria are well known advantageous soil microbes that can influence plant growth directly or indirectly (Vacheron et al., 2013). They can have direct influence on plant ability to fix atmospheric nitrogen and make better use of potassium and phosphates (Sampaio et al., 2021). In this way, they work as growth regulators, encouraging better root growth, improved seed germination and higher yield (Glick, 2012; Rehman et al., 2020). Rhizobacteria influence plants by indirectly enabling their natural flexibility to better protect against phytopathogens (Nayak et al., 2020). Among them *Bacillus* and *Pseudomonas* genera are more significant due to their broad spectrum antagonistic properties against a range of phytopathogens (Kim et al., 2011a).

**FIGURE 3:**
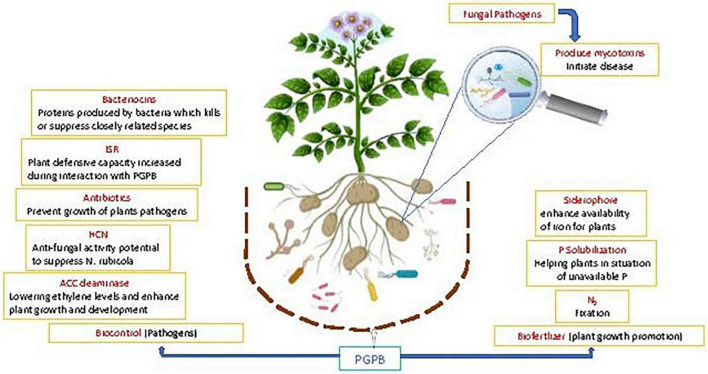
Schematic representation of the beneficial potential of PGPB in plant growth promotion and biological control of diseases: greater focus on fungal disease aspect.

In addition, PGPR exhibit pathogen antagonism (Beneduzi et al., 2012) directly and indirectly by ISR (Kloepper and Ryu, 2006). Antagonism of PGPR can be determined by their ability to generate antibiotic metabolites, such as surfactin, iturin, bacillomycin, fengycin, pyrrolnitrin, pyoluteorin, kanosamine, phenazine, 2, 4 diacetylphloroglucinol (DAPG), pyocyanin, hydrogen cyanide, and viscosinamide (Meyer et al., 2016; Prabhukarthikeyan et al., 2018). An ISR related biocontrol agent against diseases has been developed as a key tool in crop protection; the plants are more resistant to pathogen outbreak (Manikandan and Raguchander, 2014). These induced defense responses are controlled by interrelated signal transduction pathways *viz*., salicylic acid (SA), jasmonic acid (JA) and ethylene (ET); which play key part in the activation of defense linked genes (Ghanta et al., 2014) (Figure 3).

### Use of biologicals and synthetic fertilizer

The ability of *N. rubicola* to remain viable for longer periods and cause infection needs investigation and development of a proper management strategy. Currently an integrated approach involving cultural, biological, and pre-storage treatments with fungicides has been practiced (Frost et al., 2013; Liu et al., 2020). In previous decades, potato production primarily relied on use of high yielding cultivars and on the application of large quantities of synthetic fertilizers and pesticides. Despite all possible protection measures, 20–40% of global potato production is lost to pests and diseases each year (Lee et al., 2019). Postharvest application of thiabendazole, a benzimidazole fungicide has been used extensively to manage potato diseases (Liu et al., 2019). However, many strains of *Fusarium* have become resistant to thiabendazole, resulting in higher incidence and severity of potato rot (Mejdoub-Trabelsi et al., 2020). Present agricultural and environmental scenarios demand research activity to develop non-hazardous and environmentally friendly solutions to protect plants against pathogens. The phytotoxicity and residues of fungicides are related to various plant health issues and their efficacy has not been completely reliable because of development of resistance in pathogens. All these reasons promote the need for alternative and sustainable disease management which include integrated practices that involve organic farming, biological control, resistant hybrid or transgenic crops. However, novel approaches for crop productivity with minimum reliance on pesticides need to be established. According to Boulet et al. (2021), the isolated cloacal beneficial bacteria from *Sceloporus virgatus* have the potential to control *N. rubicola* by inhibiting fungal growth as well as outcompeting for space and nutrients (Bunker et al., 2021).

The adaptability of the potato crop mostly depends upon cultivar. However, crop management practices play an important role in potato crop adaptation; management practices can be based on specific agro-ecological environment, socio-economic status, and ancestral production systems. Novel strategies in smart agriculture are innovative for optimum resource use, based on decision support tools and new monitoring technologies. Remote sensing tools and global information system (GIS) along with decision support systems (DSS) and updated agriculture technologies may add to enhanced potato production. However, collaboration between biophysical and social disciplines can help to maximize the use of resources to accelerate the production of sustainable food. Fertilizer (nitrogen, phosphorus, potassium) recommendation systems are now available on small scale models as well as satellite drones and tractors, which can be carrying spectral sensors to monitor crop nutrients (Zilberman et al., 2018). These sophisticated technologies are mainly used in high-income countries but can be adapted to large-scale and diverse management data to develop new models and to support decision-making systems in underdeveloped countries. Given current global climate change, the potato crop may be at risk of many pests and diseases, forming a major threat to sustainable potato crops. Research for the development of biocontrol is very active and is expected to develop significantly in the coming years, but there are still few achievements in this field as specific management tools (decision support tools) that are missing (Thompson et al., 2017). The International Potato Center (CIP) and the European Association for Potato Research (EAPR) are coordinating in Latin America and Europe to promote biocontrol and evaluate the performance of biocontrol agents using specific procedures (Devaux et al., 2017).

To improve crop health, DSS and portable molecular diagnostic tools for early warning, monitoring, input use efficiency and control of pests and diseases can contribute to better crop production. Management strategies of farmers largely depends on application of commercial fertilizers, pesticides, and cultural practices. However, in the last few years, there has been a modification in the behavior of people toward the use of pesticides and chemical-based fertilizers, due to their role in environmental contamination. These pesticides have negative effects on human health and on the global ecosystem. The drawbacks of chemical fungicides have led to exploration of eco-friendly biological and cultural management strategies in IDM. Due to increasing food-safety awareness among consumers, scientists are looking for alternative biological control measures for plant diseases that could help to constrain use of chemicals in agriculture (Kim et al., 2011b; Ab Rahman et al., 2017). Breeding of disease resistance (R) genes is difficult but safe and an economical approach to manage the diseases (Pandolfi et al., 2017). Some natural sources, such as allelopathic plants and microbes have antagonistic potential against these fungi (Schandry and Becker, 2020; Shah et al., 2021).

Natural management of diseases and pests is a key factor in potato agro-ecosystems. In organic farming, it has been shown that the implementation of natural antagonist relationships can significantly enhance the management of potato crop pests (Crowder and Krysan, 2016). Ecosystems can also be managed by the provision of nitrogen through biological fixation and mineralization, which can be improved by practices such as legume-based intercropping, cover crops and addition of organic soil before the cultivation of potato crops. The management of ecosystems at the landscape level and in the field can deliver a series of production assistances and lessen the requirement of on-farm inputs (Firth et al., 2020). One of the most profound findings in biological investigation from the past decade revealed that, holobiont may promote plant growth, assist for abiotic tolerance, and expedite defense against phytopathogens (Backer et al., 2018; John et al., 2020; Lu et al., 2021).

Extensive manuring and treatment with mineral fertilizers are required for better potato yield, which increases production costs (Kafle et al., 2019; Du et al., 2020). Sole and excessive use of chemical fertilizers to support intensive cropping systems has a negative impact on the environment by causing long-term soil infertility, ground water contamination, green-house gas emissions and associated climate change effects and imbalance of soil and water ecosystems (Wittwer et al., 2017; Du et al., 2020). Nutrient management through integration of inorganic fertilizers and rhizobacteria (plant growth promoting rhizobacteria-PGPR) offers beneficial effects over sole application of chemical fertilizers (Sood et al., 2018; Etesami and Adl, 2020; Rasool et al., 2021). Rhizobacteria can produce mineral nutrients through natural processes of biological nitrogen fixation, phosphate solubilization and stimulates plant growth through the synthesis of plant growth promoting substances (Benaissa, 2019; Aasfar et al., 2021).

## Conclusion and future directions

Globally, change in agro-climatic conditions and indiscriminate use of pesticides have increased pathogen virulence and infestation. While increasing population is associated with decreasing availability of land for potato cultivation, increasing pathogen resistance to pesticides is worsening the situation and may lead to disruptions in global food supply. Fertile soils are rich in microbial community diversity including fungi and bacteria. Due to monocropping nature of potato, it creates imbalance of soil microbial community which increase susceptibility of plants when a new pathogen emerges, such as *N. rubicola*. To avoid the huge drawbacks of monoculture it is essential to precisely screen the available potato cultivars and newly developed germplasm against destructive pathogens such as *N. rubicola* necessitating a ranking for susceptibility and resistance. Use of certified disease-free seed, prevent tubers from the injury during harvesting, packing and transportation, and ensuring optimum storage conditions are another important consideration in pathogen cycle management. The use of pesticides further increases imbalance of microbial community in the soil, which could worsen the problem. Therefore combination of PGPR with appropriate dosages of fertilizer would be a sound solution to replenish microbial imbalance and induce plant systematic resistance.

## Author contributions

MR: conceptualize the study, formal analysis, methodology and writing—original draft and manuscript. SA: review, editing, and technical input. SK: supervision, review, editing, and technical input. NA: supervise, review, and edit the manuscript, provide technical guidelines during the study. LM: assistance in graphical presentation and technical review. MA: technical input and edited the manuscript. DS: review and editing, supervision, and technical input. All authors contributed to the article and approved the submitted version.
